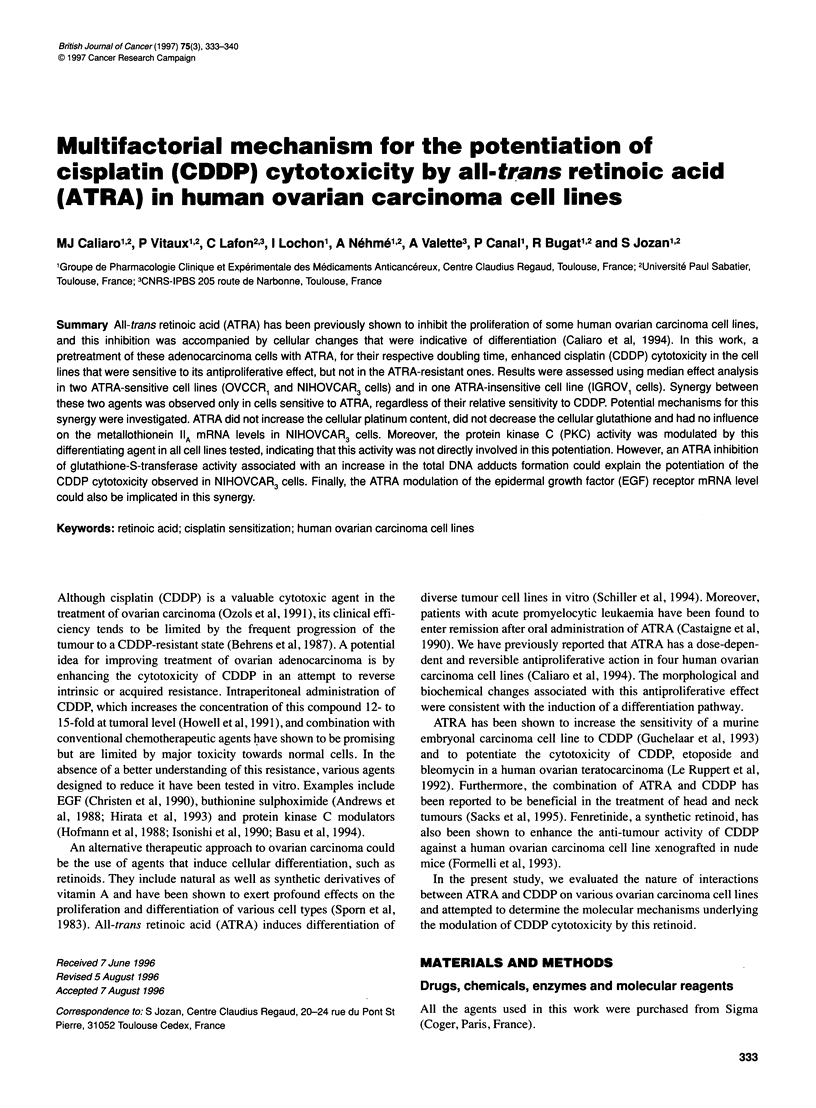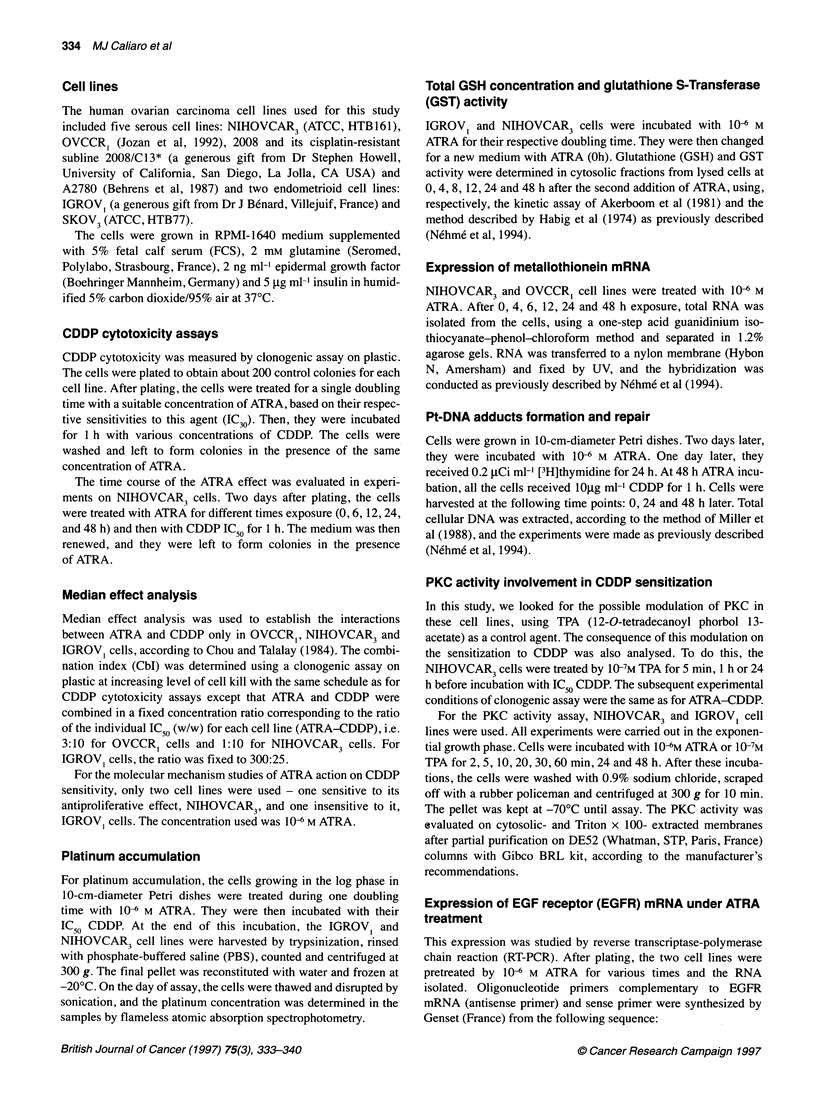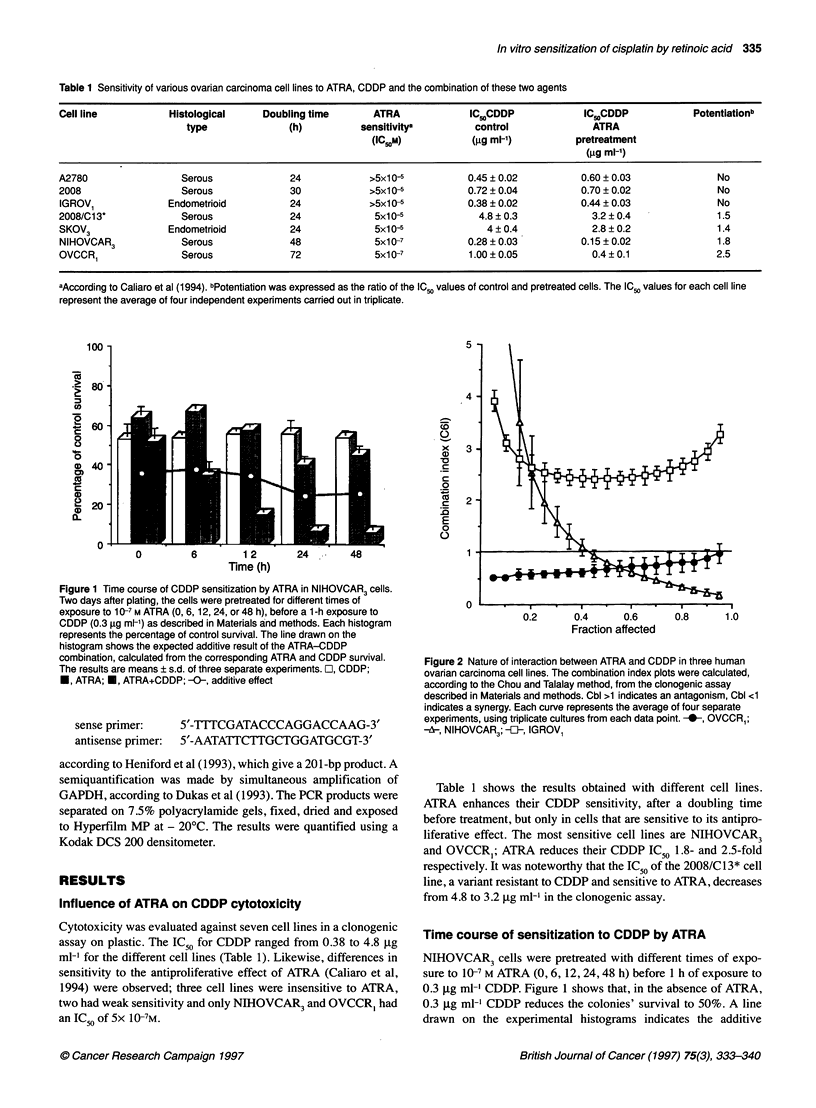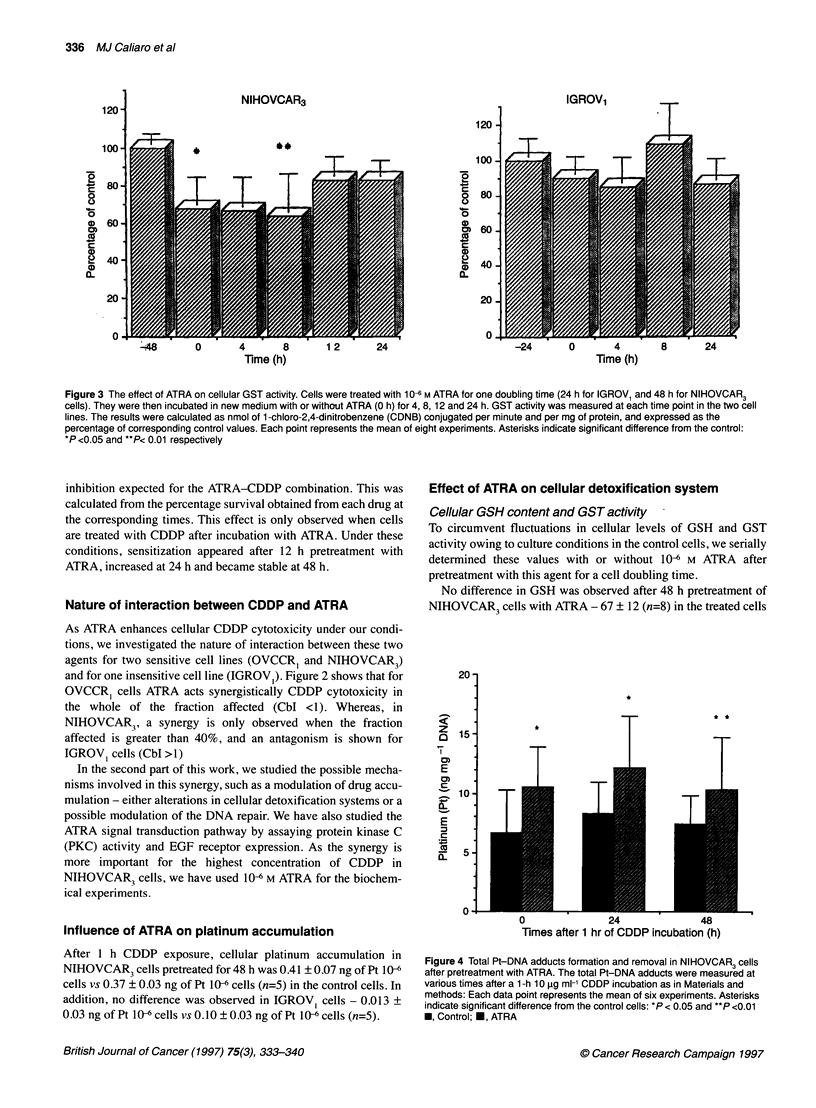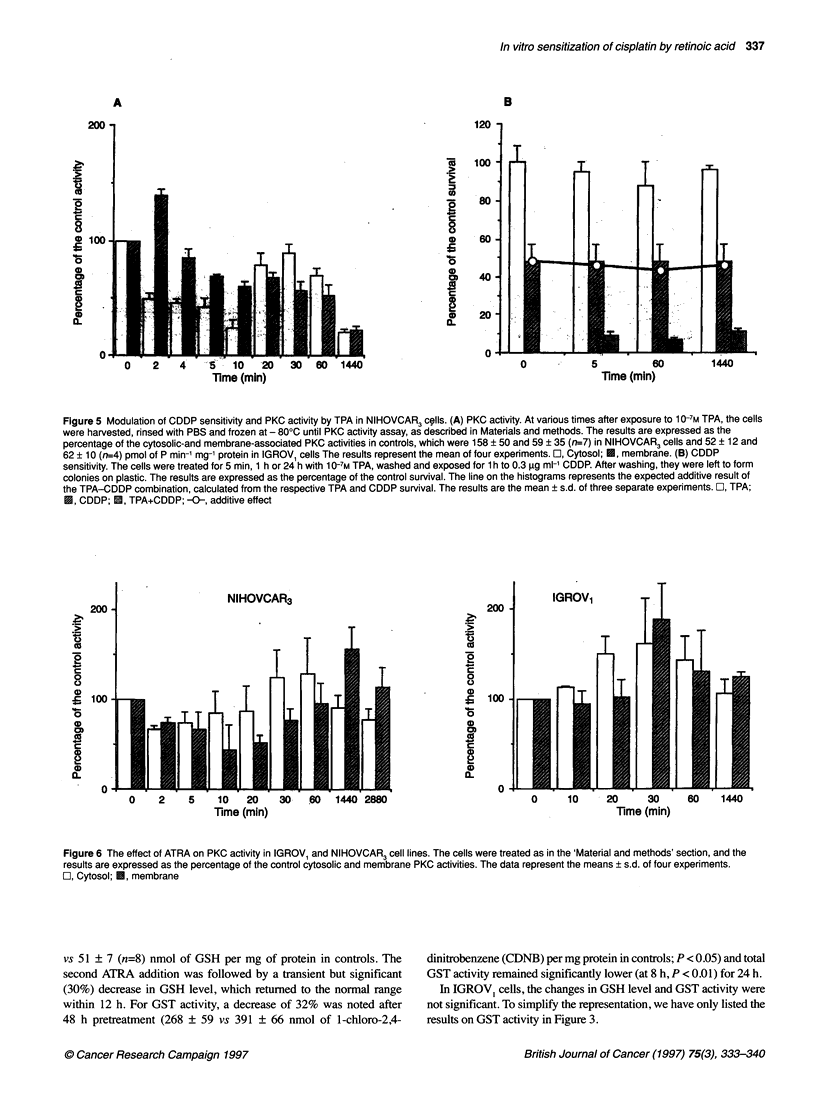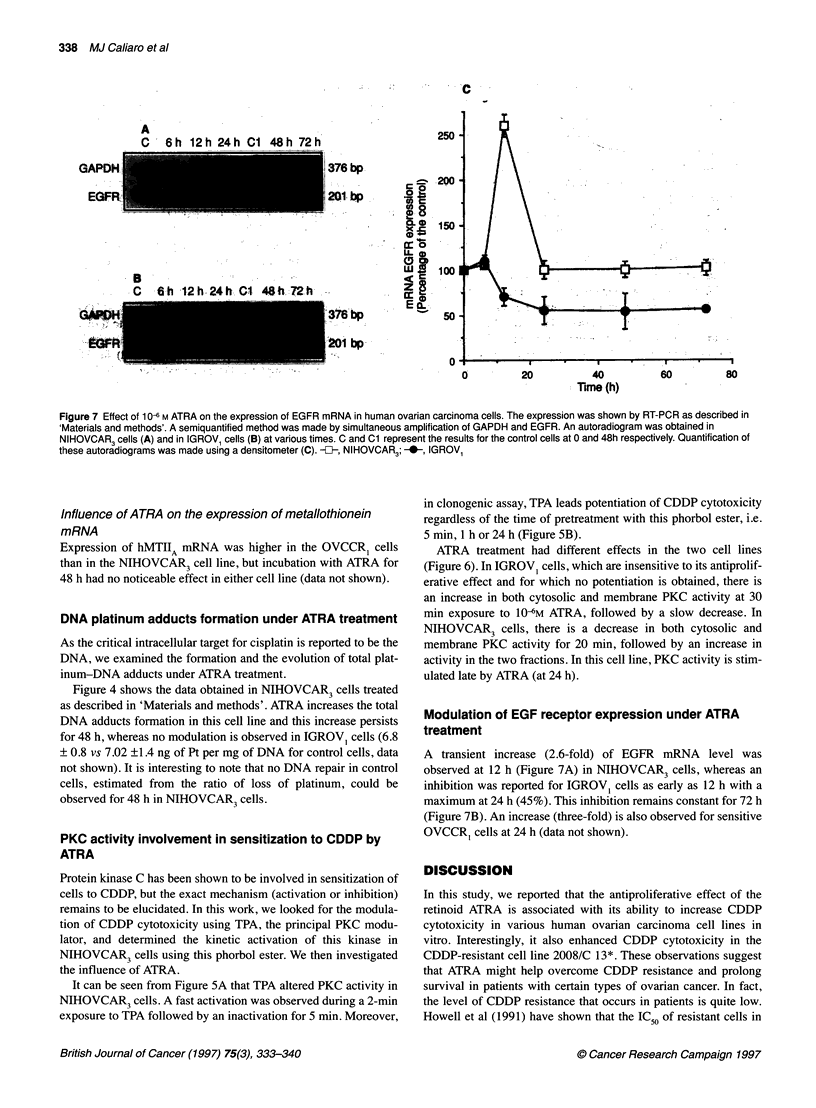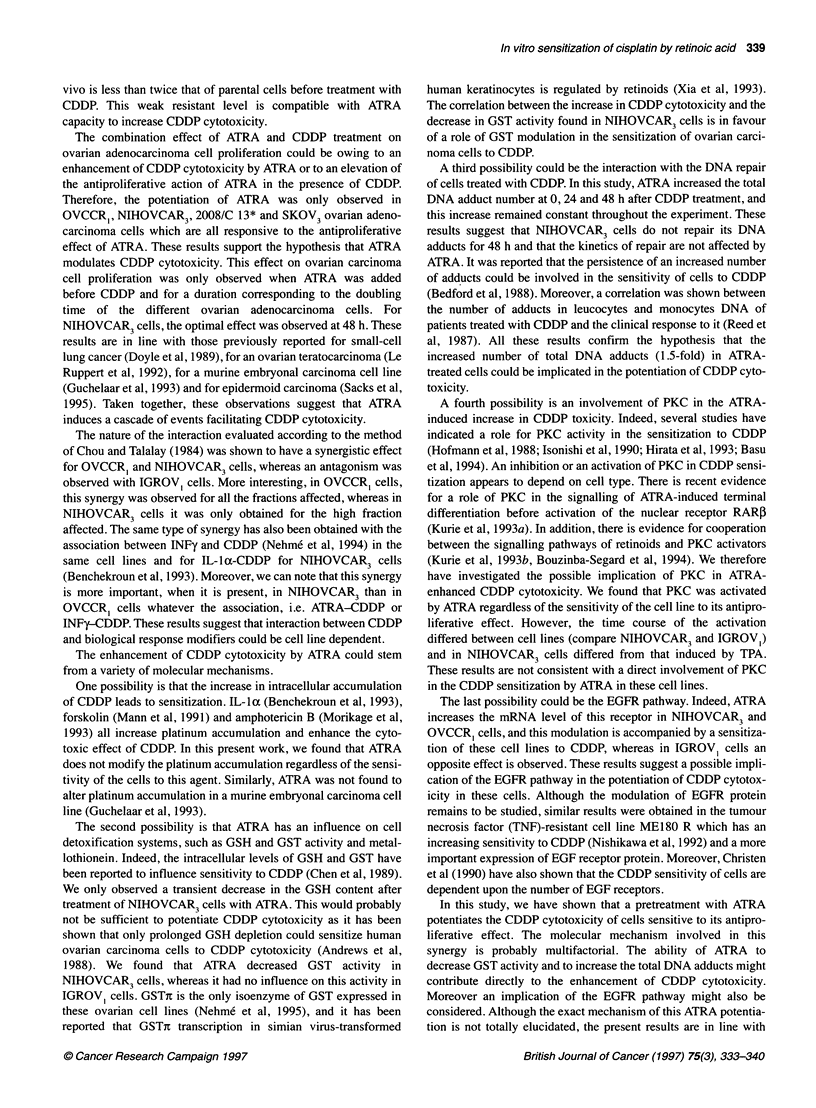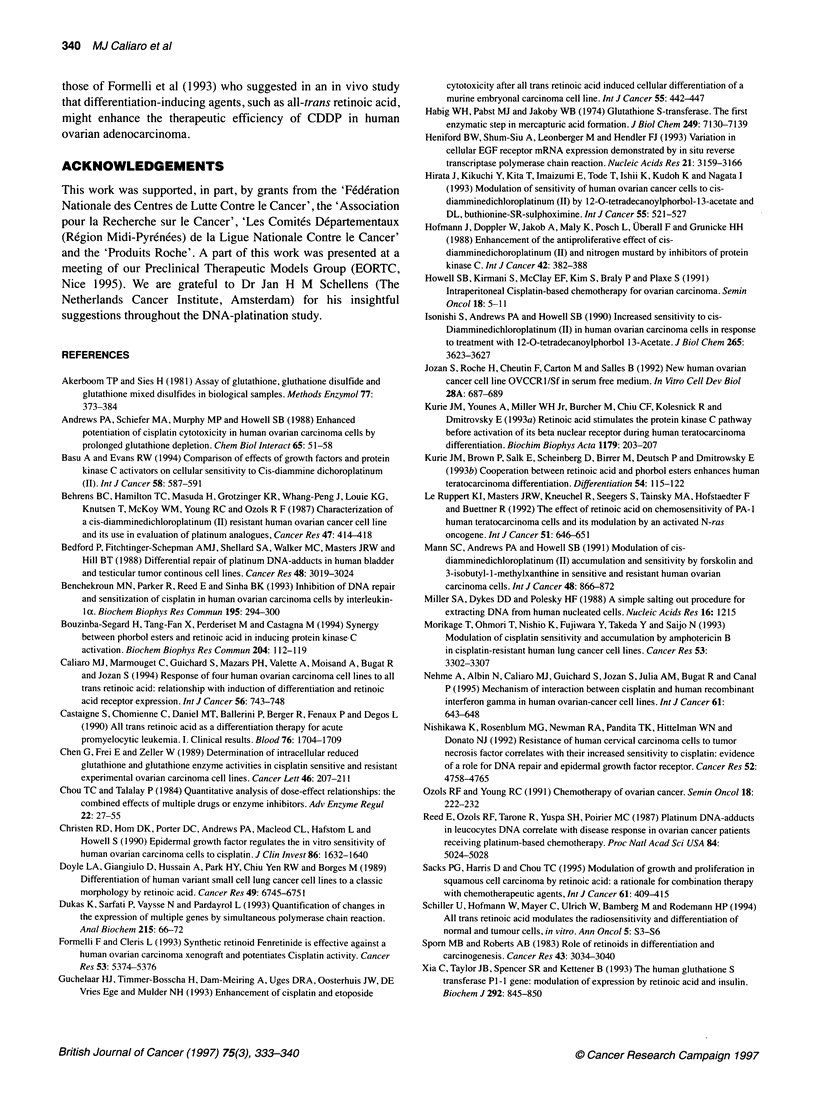# Multifactorial mechanism for the potentiation of cisplatin (CDDP) cytotoxicity by all-trans retinoic acid (ATRA) in human ovarian carcinoma cell lines.

**DOI:** 10.1038/bjc.1997.55

**Published:** 1997

**Authors:** M. J. Caliaro, P. Vitaux, C. Lafon, I. Lochon, A. Néhmé, A. Valette, P. Canal, R. Bugat, S. Jozan

**Affiliations:** Groupe de Pharmacologie Clinique et Expérimentale des Médicaments Anticancéreux, Centre Claudius Regaud, Toulouse, France.

## Abstract

**Images:**


British Journal of Cancer (1997) 75(3), 333–340
© 1997 Cancer Research Campaign

### Multifactorial mechanism for the potentiation of cisplatin (CDDP) cytotoxicity by all-*trans* retinoic acid (ATRA) in human ovarian carcinoma cell lines

**MJ Caliaro[1,2], P Vitaux[1,2], C Lafon[2,3], I Lochon[1], A Néhmé[1,2], A Valette[3], P Canal[1], R Bugat[1,2] and S Jozan[1,2]**

[1]Groupe de Pharmacologie Clinique et Expérimentale des Médicaments Anticancéreux, Centre Claudius Regaud, Toulouse, France; [2]Université Paul Sabatier, Toulouse, France; [3]CNRS-IPBS 205 route de Narbonne, Toulouse, France

**Summary** All-*trans* retinoic acid (ATRA) has been previously shown to inhibit the proliferation of some human ovarian carcinoma cell lines, and this inhibition was accompanied by cellular changes that were indicative of differentiation (Caliaro et al, 1994). In this work, a pretreatment of these adenocarcinoma cells with ATRA, for their respective doubling time, enhanced cisplatin (CDDP) cytotoxicity in the cell lines that were sensitive to its antiproliferative effect, but not in the ATRA-resistant ones. Results were assessed using median effect analysis in two ATRA-sensitive cell lines ($OVCCR_1$ and $NIHOVCAR_3$ cells) and in one ATRA-insensitive cell line ($IGROV_1$ cells). Synergy between these two agents was observed only in cells sensitive to ATRA, regardless of their relative sensitivity to CDDP. Potential mechanisms for this synergy were investigated. ATRA did not increase the cellular platinum content, did not decrease the cellular glutathione and had no influence on the metallothionein $II_A$ mRNA levels in $NIHOVCAR_3$ cells. Moreover, the protein kinase C (PKC) activity was modulated by this differentiating agent in all cell lines tested, indicating that this activity was not directly involved in this potentiation. However, an ATRA inhibition of glutathione-S-transferase activity associated with an increase in the total DNA adducts formation could explain the potentiation of the CDDP cytotoxicity observed in $NIHOVCAR_3$ cells. Finally, the ATRA modulation of the epidermal growth factor (EGF) receptor mRNA level could also be implicated in this synergy.

**Keywords:** retinoic acid; cisplatin sensitization; human ovarian carcinoma cell lines

Although cisplatin (CDDP) is a valuable cytotoxic agent in the treatment of ovarian carcinoma (Ozols et al, 1991), its clinical efficiency tends to be limited by the frequent progression of the tumour to a CDDP-resistant state (Behrens et al, 1987). A potential idea for improving treatment of ovarian adenocarcinoma is by enhancing the cytotoxicity of CDDP in an attempt to reverse intrinsic or acquired resistance. Intraperitoneal administration of CDDP, which increases the concentration of this compound 12- to 15-fold at tumoral level (Howell et al, 1991), and combination with conventional chemotherapeutic agents have shown to be promising but are limited by major toxicity towards normal cells. In the absence of a better understanding of this resistance, various agents designed to reduce it have been tested in vitro. Examples include EGF (Christen et al, 1990), buthionine sulphoximide (Andrews et al, 1988; Hirata et al, 1993) and protein kinase C modulators (Hofmann et al, 1988; Isonishi et al, 1990; Basu et al, 1994).

An alternative therapeutic approach to ovarian carcinoma could be the use of agents that induce cellular differentiation, such as retinoids. They include natural as well as synthetic derivatives of vitamin A and have been shown to exert profound effects on the proliferation and differentiation of various cell types (Sporn et al, 1983). All-*trans* retinoic acid (ATRA) induces differentiation of diverse tumour cell lines in vitro (Schiller et al, 1994). Moreover, patients with acute promyelocytic leukaemia have been found to enter remission after oral administration of ATRA (Castaigne et al, 1990). We have previously reported that ATRA has a dose-dependent and reversible antiproliferative action in four human ovarian carcinoma cell lines (Caliaro et al, 1994). The morphological and biochemical changes associated with this antiproliferative effect were consistent with the induction of a differentiation pathway.

ATRA has been shown to increase the sensitivity of a murine embryonal carcinoma cell line to CDDP (Guchelaar et al, 1993) and to potentiate the cytotoxicity of CDDP, etoposide and bleomycin in a human ovarian teratocarcinoma (Le Ruppert et al, 1992). Furthermore, the combination of ATRA and CDDP has been reported to be beneficial in the treatment of head and neck tumours (Sacks et al, 1995). Fenretinide, a synthetic retinoid, has also been shown to enhance the anti-tumour activity of CDDP against a human ovarian carcinoma cell line xenografted in nude mice (Formelli et al, 1993).

In the present study, we evaluated the nature of interactions between ATRA and CDDP on various ovarian carcinoma cell lines and attempted to determine the molecular mechanisms underlying the modulation of CDDP cytotoxicity by this retinoid.

*Received 7 June 1996*
*Revised 5 August 1996*
*Accepted 7 August 1996*

*Correspondence to:* S Jozan, Centre Claudius Regaud, 20–24 rue du Pont St Pierre, 31052 Toulouse Cedex, France

### MATERIALS AND METHODS

### Drugs, chemicals, enzymes and molecular reagents

All the agents used in this work were purchased from Sigma (Coger, Paris, France).

### Cell lines

The human ovarian carcinoma cell lines used for this study included five serous cell lines: $NIHOVCAR_3$ (ATCC, HTB161), $OVCCR_1$ (Jozan et al, 1992), 2008 and its cisplatin-resistant subline 2008/C13* (a generous gift from Dr Stephen Howell, University of California, San Diego, La Jolla, CA USA) and A2780 (Behrens et al, 1987) and two endometrioid cell lines: $IGROV_1$ (a generous gift from Dr J Bénard, Villejuif, France) and $SKOV_3$ (ATCC, HTB77).

The cells were grown in RPMI-1640 medium supplemented with 5% fetal calf serum (FCS), 2 mM glutamine (Seromed, Polylabo, Strasbourg, France), 2 ng $ml^{-1}$ epidermal growth factor (Boehringer Mannheim, Germany) and 5 µg $ml^{-1}$ insulin in humidified 5% carbon dioxide/95% air at 37°C.

### CDDP cytotoxicity assays

CDDP cytotoxicity was measured by clonogenic assay on plastic. The cells were plated to obtain about 200 control colonies for each cell line. After plating, the cells were treated for a single doubling time with a suitable concentration of ATRA, based on their respective sensitivities to this agent ($IC_{30}$). Then, they were incubated for 1 h with various concentrations of CDDP. The cells were washed and left to form colonies in the presence of the same concentration of ATRA.

The time course of the ATRA effect was evaluated in experiments on $NIHOVCAR_3$ cells. Two days after plating, the cells were treated with ATRA for different times exposure (0, 6, 12, 24, and 48 h) and then with CDDP $IC_{50}$ for 1 h. The medium was then renewed, and they were left to form colonies in the presence of ATRA.

### Median effect analysis

Median effect analysis was used to establish the interactions between ATRA and CDDP only in $OVCCR_1$, $NIHOVCAR_3$ and $IGROV_1$ cells, according to Chou and Talalay (1984). The combination index (CbI) was determined using a clonogenic assay on plastic at increasing level of cell kill with the same schedule as for CDDP cytotoxicity assays except that ATRA and CDDP were combined in a fixed concentration ratio corresponding to the ratio of the individual $IC_{50}$ (w/w) for each cell line (ATRA–CDDP), i.e. 3:10 for $OVCCR_1$ cells and 1:10 for $NIHOVCAR_3$ cells. For $IGROV_1$ cells, the ratio was fixed to 300:25.

For the molecular mechanism studies of ATRA action on CDDP sensitivity, only two cell lines were used – one sensitive to its antiproliferative effect, $NIHOVCAR_3$, and one insensitive to it, $IGROV_1$ cells. The concentration used was $10^{-6}$ M ATRA.

### Platinum accumulation

For platinum accumulation, the cells growing in the log phase in 10-cm-diameter Petri dishes were treated during one doubling time with $10^{-6}$ M ATRA. They were then incubated with their $IC_{50}$ CDDP. At the end of this incubation, the $IGROV_1$ and $NIHOVCAR_3$ cell lines were harvested by trypsinization, rinsed with phosphate-buffered saline (PBS), counted and centrifuged at 300 ***g***. The final pellet was reconstituted with water and frozen at –20°C. On the day of assay, the cells were thawed and disrupted by sonication, and the platinum concentration was determined in the samples by flameless atomic absorption spectrophotometry.

### Total GSH concentration and glutathione S-Transferase (GST) activity

$IGROV_1$ and $NIHOVCAR_3$ cells were incubated with $10^{-6}$ M ATRA for their respective doubling time. They were then changed for a new medium with ATRA (0h). Glutathione (GSH) and GST activity were determined in cytosolic fractions from lysed cells at 0, 4, 8, 12, 24 and 48 h after the second addition of ATRA, using, respectively, the kinetic assay of Akerboom et al (1981) and the method described by Habig et al (1974) as previously described (Néhmé et al, 1994).

### Expression of metallothionein mRNA

$NIHOVCAR_3$ and $OVCCR_1$ cell lines were treated with $10^{-6}$ M ATRA. After 0, 4, 6, 12, 24 and 48 h exposure, total RNA was isolated from the cells, using a one-step acid guanidinium isothiocyanate–phenol–chloroform method and separated in 1.2% agarose gels. RNA was transferred to a nylon membrane (Hybon N, Amersham) and fixed by UV, and the hybridization was conducted as previously described by Néhmé et al (1994).

### Pt-DNA adducts formation and repair

Cells were grown in 10-cm-diameter Petri dishes. Two days later, they were incubated with $10^{-6}$ M ATRA. One day later, they received 0.2 µCi $ml^{-1}$ [$^3$H]thymidine for 24 h. At 48 h ATRA incubation, all the cells received 10µg $ml^{-1}$ CDDP for 1 h. Cells were harvested at the following time points: 0, 24 and 48 h later. Total cellular DNA was extracted, according to the method of Miller et al (1988), and the experiments were made as previously described (Néhmé et al, 1994).

### PKC activity involvement in CDDP sensitization

In this study, we looked for the possible modulation of PKC in these cell lines, using TPA (12-*O*-tetradecanoyl phorbol 13-acetate) as a control agent. The consequence of this modulation on the sensitization to CDDP was also analysed. To do this, the $NIHOVCAR_3$ cells were treated by $10^{-7}$M TPA for 5 min, 1 h or 24 h before incubation with $IC_{50}$ CDDP. The subsequent experimental conditions of clonogenic assay were the same as for ATRA–CDDP.

For the PKC activity assay, $NIHOVCAR_3$ and $IGROV_1$ cell lines were used. All experiments were carried out in the exponential growth phase. Cells were incubated with $10^{-6}$M ATRA or $10^{-7}$M TPA for 2, 5, 10, 20, 30, 60 min, 24 and 48 h. After these incubations, the cells were washed with 0.9% sodium chloride, scraped off with a rubber policeman and centrifuged at 300 ***g*** for 10 min. The pellet was kept at –70°C until assay. The PKC activity was evaluated on cytosolic- and Triton × 100- extracted membranes after partial purification on DE52 (Whatman, STP, Paris, France) columns with Gibco BRL kit, according to the manufacturer's recommendations.

### Expression of EGF receptor (EGFR) mRNA under ATRA treatment

This expression was studied by reverse transcriptase-polymerase chain reaction (RT-PCR). After plating, the two cell lines were pretreated by $10^{-6}$ M ATRA for various times and the RNA isolated. Oligonucleotide primers complementary to EGFR mRNA (antisense primer) and sense primer were synthesized by Genset (France) from the following sequence:

© Cancer Research Campaign 1997

**Table 1** Sensitivity of various ovarian carcinoma cell lines to ATRA, CDDP and the combination of these two agents

| Cell line | Histological type | Doubling time (h) | ATRA sensitivity[a] ($IC_{50}$M) | $IC_{50}$CDDP control (μg $ml^{-1}$) | $IC_{50}$CDDP ATRA pretreatment (μg $ml^{-1}$) | Potentiation[b] |
|---|---|---|---|---|---|---|
| A2780 | Serous | 24 | >5×$10^{-5}$ | 0.45 ± 0.02 | 0.60 ± 0.03 | No |
| 2008 | Serous | 30 | >5×$10^{-5}$ | 0.72 ± 0.04 | 0.70 ± 0.02 | No |
| $IGROV_1$ | Endometrioid | 24 | >5×$10^{-5}$ | 0.38 ± 0.02 | 0.44 ± 0.03 | No |
| 2008/C13* | Serous | 24 | 5×$10^{-5}$ | 4.8 ± 0.3 | 3.2 ± 0.4 | 1.5 |
| $SKOV_3$ | Endometrioid | 24 | 5×$10^{-5}$ | 4 ± 0.4 | 2.8 ± 0.2 | 1.4 |
| $NIHOVCAR_3$ | Serous | 48 | 5×$10^{-7}$ | 0.28 ± 0.03 | 0.15 ± 0.02 | 1.8 |
| $OVCCR_1$ | Serous | 72 | 5×$10^{-7}$ | 1.00 ± 0.05 | 0.4 ± 0.1 | 2.5 |

[a]According to Caliaro et al (1994). [b]Potentiation was expressed as the ratio of the $IC_{50}$ values of control and pretreated cells. The $IC_{50}$ values for each cell line represent the average of four independent experiments carried out in triplicate.

**Figure 1** Time course of CDDP sensitization by ATRA in $NIHOVCAR_3$ cells. Two days after plating, the cells were pretreated for different times of exposure to $10^{-7}$ M ATRA (0, 6, 12, 24, or 48 h), before a 1-h exposure to CDDP (0.3 μg $ml^{-1}$) as described in Materials and methods. Each histogram represents the percentage of control survival. The line drawn on the histogram shows the expected additive result of the ATRA–CDDP combination, calculated from the corresponding ATRA and CDDP survival. The results are means ± s.d. of three separate experiments. □, CDDP; ■, ATRA; ■, ATRA+CDDP; –O–, additive effect

**Figure 2** Nature of interaction between ATRA and CDDP in three human ovarian carcinoma cell lines. The combination index plots were calculated, according to the Chou and Talalay method, from the clonogenic assay described in Materials and methods. CbI >1 indicates an antagonism, CbI <1 indicates a synergy. Each curve represents the average of four separate experiments, using triplicate cultures from each data point. –●–, $OVCCR_1$; –△–, $NIHOVCAR_3$; –□–, $IGROV_1$

| | |
|---|---|
| sense primer: | 5′-TTTCGATACCCAGGACCAAG-3′ |
| antisense primer: | 5′-AATATTCTTGCTGGATGCGT-3′ |

according to Heniford et al (1993), which give a 201-bp product. A semiquantification was made by simultaneous amplification of GAPDH, according to Dukas et al (1993). The PCR products were separated on 7.5% polyacrylamide gels, fixed, dried and exposed to Hyperfilm MP at – 20°C. The results were quantified using a Kodak DCS 200 densitometer.

### RESULTS

### Influence of ATRA on CDDP cytotoxicity

Cytotoxicity was evaluated against seven cell lines in a clonogenic assay on plastic. The $IC_{50}$ for CDDP ranged from 0.38 to 4.8 μg $ml^{-1}$ for the different cell lines (Table 1). Likewise, differences in sensitivity to the antiproliferative effect of ATRA (Caliaro et al, 1994) were observed; three cell lines were insensitive to ATRA, two had weak sensitivity and only $NIHOVCAR_3$ and $OVCCR_1$ had an $IC_{50}$ of 5× $10^{-7}$M.

Table 1 shows the results obtained with different cell lines. ATRA enhances their CDDP sensitivity, after a doubling time before treatment, but only in cells that are sensitive to its antiproliferative effect. The most sensitive cell lines are $NIHOVCAR_3$ and $OVCCR_1$; ATRA reduces their CDDP $IC_{50}$ 1.8- and 2.5-fold respectively. It was noteworthy that the $IC_{50}$ of the 2008/C13* cell line, a variant resistant to CDDP and sensitive to ATRA, decreases from 4.8 to 3.2 μg $ml^{-1}$ in the clonogenic assay.

### Time course of sensitization to CDDP by ATRA

$NIHOVCAR_3$ cells were pretreated with different times of exposure to $10^{-7}$ M ATRA (0, 6, 12, 24, 48 h) before 1 h of exposure to 0.3 μg $ml^{-1}$ CDDP. Figure 1 shows that, in the absence of ATRA, 0.3 μg $ml^{-1}$ CDDP reduces the colonies' survival to 50%. A line drawn on the experimental histograms indicates the additive

© Cancer Research Campaign 1997

**Figure 3** The effect of ATRA on cellular GST activity. Cells were treated with $10^{-6}$ M ATRA for one doubling time (24 h for $IGROV_1$ and 48 h for $NIHOVCAR_3$ cells). They were then incubated in new medium with or without ATRA (0 h) for 4, 8, 12 and 24 h. GST activity was measured at each time point in the two cell lines. The results were calculated as nmol of 1-chloro-2,4-dinitrobenzene (CDNB) conjugated per minute and per mg of protein, and expressed as the percentage of corresponding control values. Each point represents the mean of eight experiments. Asterisks indicate significant difference from the control: *$P$ <0.05 and **$P$< 0.01 respectively inhibition expected for the ATRA–CDDP combination. This was calculated from the percentage survival obtained from each drug at the corresponding times. This effect is only observed when cells are treated with CDDP after incubation with ATRA. Under these conditions, sensitization appeared after 12 h pretreatment with ATRA, increased at 24 h and became stable at 48 h.

### Nature of interaction between CDDP and ATRA

As ATRA enhances cellular CDDP cytotoxicity under our conditions, we investigated the nature of interaction between these two agents for two sensitive cell lines ($OVCCR_1$ and $NIHOVCAR_3$) and for one insensitive cell line ($IGROV_1$). Figure 2 shows that for $OVCCR_1$ cells ATRA acts synergistically CDDP cytotoxicity in the whole of the fraction affected (CbI <1). Whereas, in $NIHOVCAR_3$, a synergy is only observed when the fraction affected is greater than 40%, and an antagonism is shown for $IGROV_1$ cells (CbI >1)

In the second part of this work, we studied the possible mechanisms involved in this synergy, such as a modulation of drug accumulation – either alterations in cellular detoxification systems or a possible modulation of the DNA repair. We have also studied the ATRA signal transduction pathway by assaying protein kinase C (PKC) activity and EGF receptor expression. As the synergy is more important for the highest concentration of CDDP in $NIHOVCAR_3$ cells, we have used $10^{-6}$ M ATRA for the biochemical experiments.

### Influence of ATRA on platinum accumulation

After 1 h CDDP exposure, cellular platinum accumulation in $NIHOVCAR_3$ cells pretreated for 48 h was 0.41 ± 0.07 ng of Pt $10^{-6}$ cells *vs* 0.37 ± 0.03 ng of Pt $10^{-6}$ cells ($n$=5) in the control cells. In addition, no difference was observed in $IGROV_1$ cells – 0.013 ± 0.03 ng of Pt $10^{-6}$ cells *vs* 0.10 ± 0.03 ng of Pt $10^{-6}$ cells ($n$=5).

### Effect of ATRA on cellular detoxification system

#### *Cellular GSH content and GST activity*

To circumvent fluctuations in cellular levels of GSH and GST activity owing to culture conditions in the control cells, we serially determined these values with or without $10^{-6}$ M ATRA after pretreatment with this agent for a cell doubling time.

No difference in GSH was observed after 48 h pretreatment of $NIHOVCAR_3$ cells with ATRA – 67 ± 12 ($n$=8) in the treated cells

**Figure 4** Total Pt–DNA adducts formation and removal in $NIHOVCAR_3$ cells after pretreatment with ATRA. The total Pt–DNA adducts were measured at various times after a 1-h 10 µg $ml^{-1}$ CDDP incubation as in Materials and methods: Each data point represents the mean of six experiments. Asterisks indicate significant difference from the control cells: *$P$ < 0.05 and **$P$ <0.01 ■, Control; ▨, ATRA

© Cancer Research Campaign 1997

**Figure 5** Modulation of CDDP sensitivity and PKC activity by TPA in NIHOVCAR$_3$ cells. (**A**) PKC activity. At various times after exposure to $10^{-7}$M TPA, the cells were harvested, rinsed with PBS and frozen at – 80°C until PKC activity assay, as described in Materials and methods. The results are expressed as the percentage of the cytosolic-and membrane-associated PKC activities in controls, which were 158 ± 50 and 59 ± 35 (*n*=7) in NIHOVCAR$_3$ cells and 52 ± 12 and 62 ± 10 (*n*=4) pmol of P min$^{-1}$ mg$^{-1}$ protein in IGROV$_1$ cells The results represent the mean of four experiments. □, Cytosol; ▨, membrane. (**B**) CDDP sensitivity. The cells were treated for 5 min, 1 h or 24 h with $10^{-7}$M TPA, washed and exposed for 1h to 0.3 µg ml$^{-1}$ CDDP. After washing, they were left to form colonies on plastic. The results are expressed as the percentage of the control survival. The line on the histograms represents the expected additive result of the TPA–CDDP combination, calculated from the respective TPA and CDDP survival. The results are the mean ± s.d. of three separate experiments. □, TPA; ▨, CDDP; ▩, TPA+CDDP; –O–, additive effect

**Figure 6** The effect of ATRA on PKC activity in IGROV$_1$ and NIHOVCAR$_3$ cell lines. The cells were treated as in the 'Material and methods' section, and the results are expressed as the percentage of the control cytosolic and membrane PKC activities. The data represent the means ± s.d. of four experiments. □, Cytosol; ▨, membrane

*vs* 51 ± 7 (*n*=8) nmol of GSH per mg of protein in controls. The second ATRA addition was followed by a transient but significant (30%) decrease in GSH level, which returned to the normal range within 12 h. For GST activity, a decrease of 32% was noted after 48 h pretreatment (268 ± 59 *vs* 391 ± 66 nmol of 1-chloro-2,4-dinitrobenzene (CDNB) per mg protein in controls; $P < 0.05$) and total GST activity remained significantly lower (at 8 h, $P < 0.01$) for 24 h.

In IGROV$_1$ cells, the changes in GSH level and GST activity were not significant. To simplify the representation, we have only listed the results on GST activity in Figure 3.

© Cancer Research Campaign 1997

**Figure 7** Effect of $10^{-6}$ M ATRA on the expression of EGFR mRNA in human ovarian carcinoma cells. The expression was shown by RT-PCR as described in 'Materials and methods'. A semiquantified method was made by simultaneous amplification of GAPDH and EGFR. An autoradiogram was obtained in $NIHOVCAR_3$ cells (**A**) and in $IGROV_1$ cells (**B**) at various times. C and C1 represent the results for the control cells at 0 and 48h respectively. Quantification of these autoradiograms was made using a densitometer (**C**). -□-, $NIHOVCAR_3$; -●-, $IGROV_1$

### *Influence of ATRA on the expression of metallothionein mRNA*

Expression of $hMTII_A$ mRNA was higher in the $OVCCR_1$ cells than in the $NIHOVCAR_3$ cell line, but incubation with ATRA for 48 h had no noticeable effect in either cell line (data not shown).

### DNA platinum adducts formation under ATRA treatment

As the critical intracellular target for cisplatin is reported to be the DNA, we examined the formation and the evolution of total platinum–DNA adducts under ATRA treatment.

Figure 4 shows the data obtained in $NIHOVCAR_3$ cells treated as described in 'Materials and methods'. ATRA increases the total DNA adducts formation in this cell line and this increase persists for 48 h, whereas no modulation is observed in $IGROV_1$ cells (6.8 ± 0.8 *vs* 7.02 ±1.4 ng of Pt per mg of DNA for control cells, data not shown). It is interesting to note that no DNA repair in control cells, estimated from the ratio of loss of platinum, could be observed for 48 h in $NIHOVCAR_3$ cells.

### PKC activity involvement in sensitization to CDDP by ATRA

Protein kinase C has been shown to be involved in sensitization of cells to CDDP, but the exact mechanism (activation or inhibition) remains to be elucidated. In this work, we looked for the modulation of CDDP cytotoxicity using TPA, the principal PKC modulator, and determined the kinetic activation of this kinase in $NIHOVCAR_3$ cells using this phorbol ester. We then investigated the influence of ATRA.

It can be seen from Figure 5A that TPA altered PKC activity in $NIHOVCAR_3$ cells. A fast activation was observed during a 2-min exposure to TPA followed by an inactivation for 5 min. Moreover, in clonogenic assay, TPA leads potentiation of CDDP cytotoxicity regardless of the time of pretreatment with this phorbol ester, i.e. 5 min, 1 h or 24 h (Figure 5B).

ATRA treatment had different effects in the two cell lines (Figure 6). In $IGROV_1$ cells, which are insensitive to its antiproliferative effect and for which no potentiation is obtained, there is an increase in both cytosolic and membrane PKC activity at 30 min exposure to $10^{-6}$M ATRA, followed by a slow decrease. In $NIHOVCAR_3$ cells, there is a decrease in both cytosolic and membrane PKC activity for 20 min, followed by an increase in activity in the two fractions. In this cell line, PKC activity is stimulated late by ATRA (at 24 h).

### Modulation of EGF receptor expression under ATRA treatment

A transient increase (2.6-fold) of EGFR mRNA level was observed at 12 h (Figure 7A) in $NIHOVCAR_3$ cells, whereas an inhibition was reported for $IGROV_1$ cells as early as 12 h with a maximum at 24 h (45%). This inhibition remains constant for 72 h (Figure 7B). An increase (three-fold) is also observed for sensitive $OVCCR_1$ cells at 24 h (data not shown).

### DISCUSSION

In this study, we reported that the antiproliferative effect of the retinoid ATRA is associated with its ability to increase CDDP cytotoxicity in various human ovarian carcinoma cell lines in vitro. Interestingly, it also enhanced CDDP cytotoxicity in the CDDP-resistant cell line 2008/C 13*. These observations suggest that ATRA might help overcome CDDP resistance and prolong survival in patients with certain types of ovarian cancer. In fact, the level of CDDP resistance that occurs in patients is quite low. Howell et al (1991) have shown that the $IC_{50}$ of resistant cells in

© Cancer Research Campaign 1997

vivo is less than twice that of parental cells before treatment with CDDP. This weak resistant level is compatible with ATRA capacity to increase CDDP cytotoxicity.

The combination effect of ATRA and CDDP treatment on ovarian adenocarcinoma cell proliferation could be owing to an enhancement of CDDP cytotoxicity by ATRA or to an elevation of the antiproliferative action of ATRA in the presence of CDDP. Therefore, the potentiation of ATRA was only observed in $OVCCR_1$, $NIHOVCAR_3$, 2008/C 13* and $SKOV_3$ ovarian adenocarcinoma cells which are all responsive to the antiproliferative effect of ATRA. These results support the hypothesis that ATRA modulates CDDP cytotoxicity. This effect on ovarian carcinoma cell proliferation was only observed when ATRA was added before CDDP and for a duration corresponding to the doubling time of the different ovarian adenocarcinoma cells. For $NIHOVCAR_3$ cells, the optimal effect was observed at 48 h. These results are in line with those previously reported for small-cell lung cancer (Doyle et al, 1989), for an ovarian teratocarcinoma (Le Ruppert et al, 1992), for a murine embryonal carcinoma cell line (Guchelaar et al, 1993) and for epidermoid carcinoma (Sacks et al, 1995). Taken together, these observations suggest that ATRA induces a cascade of events facilitating CDDP cytotoxicity.

The nature of the interaction evaluated according to the method of Chou and Talalay (1984) was shown to have a synergistic effect for $OVCCR_1$ and $NIHOVCAR_3$ cells, whereas an antagonism was observed with $IGROV_1$ cells. More interesting, in $OVCCR_1$ cells, this synergy was observed for all the fractions affected, whereas in $NIHOVCAR_3$ cells it was only obtained for the high fraction affected. The same type of synergy has also been obtained with the association between INFγ and CDDP (Nehmé et al, 1994) in the same cell lines and for IL-1α-CDDP for $NIHOVCAR_3$ cells (Benchekroun et al, 1993). Moreover, we can note that this synergy is more important, when it is present, in $NIHOVCAR_3$ than in $OVCCR_1$ cells whatever the association, i.e. ATRA–CDDP or INFγ–CDDP. These results suggest that interaction between CDDP and biological response modifiers could be cell line dependent.

The enhancement of CDDP cytotoxicity by ATRA could stem from a variety of molecular mechanisms.

One possibility is that the increase in intracellular accumulation of CDDP leads to sensitization. IL-1α (Benchekroun et al, 1993), forskolin (Mann et al, 1991) and amphotericin B (Morikage et al, 1993) all increase platinum accumulation and enhance the cytotoxic effect of CDDP. In this present work, we found that ATRA does not modify the platinum accumulation regardless of the sensitivity of the cells to this agent. Similarly, ATRA was not found to alter platinum accumulation in a murine embryonal carcinoma cell line (Guchelaar et al, 1993).

The second possibility is that ATRA has an influence on cell detoxification systems, such as GSH and GST activity and metallothionein. Indeed, the intracellular levels of GSH and GST have been reported to influence sensitivity to CDDP (Chen et al, 1989). We only observed a transient decrease in the GSH content after treatment of $NIHOVCAR_3$ cells with ATRA. This would probably not be sufficient to potentiate CDDP cytotoxicity as it has been shown that only prolonged GSH depletion could sensitize human ovarian carcinoma cells to CDDP cytotoxicity (Andrews et al, 1988). We found that ATRA decreased GST activity in $NIHOVCAR_3$ cells, whereas it had no influence on this activity in $IGROV_1$ cells. GSTπ is the only isoenzyme of GST expressed in these ovarian cell lines (Nehmé et al, 1995), and it has been reported that GSTπ transcription in simian virus-transformed human keratinocytes is regulated by retinoids (Xia et al, 1993). The correlation between the increase in CDDP cytotoxicity and the decrease in GST activity found in $NIHOVCAR_3$ cells is in favour of a role of GST modulation in the sensitization of ovarian carcinoma cells to CDDP.

A third possibility could be the interaction with the DNA repair of cells treated with CDDP. In this study, ATRA increased the total DNA adduct number at 0, 24 and 48 h after CDDP treatment, and this increase remained constant throughout the experiment. These results suggest that $NIHOVCAR_3$ cells do not repair its DNA adducts for 48 h and that the kinetics of repair are not affected by ATRA. It was reported that the persistence of an increased number of adducts could be involved in the sensitivity of cells to CDDP (Bedford et al, 1988). Moreover, a correlation was shown between the number of adducts in leucocytes and monocytes DNA of patients treated with CDDP and the clinical response to it (Reed et al, 1987). All these results confirm the hypothesis that the increased number of total DNA adducts (1.5-fold) in ATRA-treated cells could be implicated in the potentiation of CDDP cytotoxicity.

A fourth possibility is an involvement of PKC in the ATRA-induced increase in CDDP toxicity. Indeed, several studies have indicated a role for PKC activity in the sensitization to CDDP (Hofmann et al, 1988; Isonishi et al, 1990; Hirata et al, 1993; Basu et al, 1994). An inhibition or an activation of PKC in CDDP sensitization appears to depend on cell type. There is recent evidence for a role of PKC in the signalling of ATRA-induced terminal differentiation before activation of the nuclear receptor RARβ (Kurie et al, 1993*a*). In addition, there is evidence for cooperation between the signalling pathways of retinoids and PKC activators (Kurie et al, 1993*b*, Bouzinba-Segard et al, 1994). We therefore have investigated the possible implication of PKC in ATRA-enhanced CDDP cytotoxicity. We found that PKC was activated by ATRA regardless of the sensitivity of the cell line to its antiproliferative effect. However, the time course of the activation differed between cell lines (compare $NIHOVCAR_3$ and $IGROV_1$) and in $NIHOVCAR_3$ cells differed from that induced by TPA. These results are not consistent with a direct involvement of PKC in the CDDP sensitization by ATRA in these cell lines.

The last possibility could be the EGFR pathway. Indeed, ATRA increases the mRNA level of this receptor in $NIHOVCAR_3$ and $OVCCR_1$ cells, and this modulation is accompanied by a sensitization of these cell lines to CDDP, whereas in $IGROV_1$ cells an opposite effect is observed. These results suggest a possible implication of the EGFR pathway in the potentiation of CDDP cytotoxicity in these cells. Although the modulation of EGFR protein remains to be studied, similar results were obtained in the tumour necrosis factor (TNF)-resistant cell line ME180 R which has an increasing sensitivity to CDDP (Nishikawa et al, 1992) and a more important expression of EGF receptor protein. Moreover, Christen et al (1990) have also shown that the CDDP sensitivity of cells are dependent upon the number of EGF receptors.

In this study, we have shown that a pretreatment with ATRA potentiates the CDDP cytotoxicity of cells sensitive to its antiproliferative effect. The molecular mechanism involved in this synergy is probably multifactorial. The ability of ATRA to decrease GST activity and to increase the total DNA adducts might contribute directly to the enhancement of CDDP cytotoxicity. Moreover an implication of the EGFR pathway might also be considered. Although the exact mechanism of this ATRA potentiation is not totally elucidated, the present results are in line with those of Formelli et al (1993) who suggested in an in vivo study that differentiation-inducing agents, such as all-*trans* retinoic acid, might enhance the therapeutic efficiency of CDDP in human ovarian adenocarcinoma.

### ACKNOWLEDGEMENTS

This work was supported, in part, by grants from the 'Fédération Nationale des Centres de Lutte Contre le Cancer', the 'Association pour la Recherche sur le Cancer', 'Les Comités Départementaux (Région Midi-Pyrénées) de la Ligue Nationale Contre le Cancer' and the 'Produits Roche'. A part of this work was presented at a meeting of our Preclinical Therapeutic Models Group (EORTC, Nice 1995). We are grateful to Dr Jan H M Schellens (The Netherlands Cancer Institute, Amsterdam) for his insightful suggestions throughout the DNA-platination study.

### REFERENCES

Akerboom TP and Sies H (1981) Assay of glutathione, gluthatione disulfide and glutathione mixed disulfides in biological samples. *Methods Enzymol* **77**: 373–384

Andrews PA, Schiefer MA, Murphy MP and Howell SB (1988) Enhanced potentiation of cisplatin cytotoxicity in human ovarian carcinoma cells by prolonged glutathione depletion. *Chem Biol Interact* **65**: 51–58

Basu A and Evans RW (1994) Comparison of effects of growth factors and protein kinase C activators on cellular sensitivity to Cis-diammine dichoroplatinum (II). *Int J Cancer* **58**: 587–591

Behrens BC, Hamilton TC, Masuda H, Grotzinger KR, Whang-Peng J, Louie KG, Knutsen T, McKoy WM, Young RC and Ozols R F (1987) Characterization of a cis-diamminedichloroplatinum (II) resistant human ovarian cancer cell line and its use in evaluation of platinum analogues, *Cancer Res* **47**: 414–418

Bedford P, Fitchtinger-Schepman AMJ, Shellard SA, Walker MC, Masters JRW and Hill BT (1988) Differential repair of platinum DNA-adducts in human bladder and testicular tumor continous cell lines. *Cancer Res* **48**: 3019–3024

Benchekroun MN, Parker R, Reed E and Sinha BK (1993) Inhibition of DNA repair and sensitization of cisplatin in human ovarian carcinoma cells by interleukin-1α. *Biochem Biophys Res Commun* **195**: 294–300

Bouzinba-Segard H, Tang-Fan X, Perderiset M and Castagna M (1994) Synergy between phorbol esters and retinoic acid in inducing protein kinase C activation. *Biochem Biophys Res Commun* **204**: 112–119

Caliaro MJ, Marmouget C, Guichard S, Mazars PH, Valette A, Moisand A, Bugat R and Jozan S (1994) Response of four human ovarian carcinoma cell lines to all trans retinoic acid: relationship with induction of differentiation and retinoic acid receptor expression. *Int J Cancer* **56**: 743–748

Castaigne S, Chomienne C, Daniel MT, Ballerini P, Berger R, Fenaux P and Degos L (1990) All trans retinoic acid as a differentiation therapy for acute promyelocytic leukemia. I. Clinical results. *Blood* **76**: 1704–1709

Chen G, Frei E and Zeller W (1989) Determination of intracellular reduced glutathione and glutathione enzyme activities in cisplatin sensitive and resistant experimental ovarian carcinoma cell lines. *Cancer Lett* **46**: 207–211

Chou TC and Talalay P (1984) Quantitative analysis of dose-effect relationships: the combined effects of multiple drugs or enzyme inhibitors. *Adv Enzyme Regul* **22**: 27–55

Christen RD, Hom DK, Porter DC, Andrews PA, Macleod CL, Hafstom L and Howell S (1990) Epidermal growth factor regulates the in vitro sensitivity of human ovarian carcinoma cells to cisplatin. *J Clin Invest* **86**: 1632–1640

Doyle LA, Giangiulo D, Hussain A, Park HY, Chiu Yen RW and Borges M (1989) Differentiation of human variant small cell lung cancer cell lines to a classic morphology by retinoic acid. *Cancer Res* **49**: 6745–6751

Dukas K, Sarfati P, Vaysse N and Pardayrol L (1993) Quantification of changes in the expression of multiple genes by simultaneous polymerase chain reaction. *Anal Biochem* **215**: 66–72

Formelli F and Cleris L (1993) Synthetic retinoid Fenretinide is effective against a human ovarian carcinoma xenograft and potentiates Cisplatin activity. *Cancer Res* **53**: 5374–5376

Guchelaar HJ, Timmer-Bosscha H, Dam-Meiring A, Uges DRA, Oosterhuis JW, DE Vries Ege and Mulder NH (1993) Enhancement of cisplatin and etoposide cytotoxicity after all trans retinoic acid induced cellular differentiation of a murine embryonal carcinoma cell line. *Int J Cancer* **55**: 442–447

Habig WH, Pabst MJ and Jakoby WB (1974) Glutathione S-transferase. The first enzymatic step in mercapturic acid formation. *J Biol Chem* **249**: 7130–7139

Heniford BW, Shum-Siu A, Leonberger M and Hendler FJ (1993) Variation in cellular EGF receptor mRNA expression demonstrated by in situ reverse transcriptase polymerase chain reaction. *Nucleic Acids Res* **21**: 3159–3166

Hirata J, Kikuchi Y, Kita T, Imaizumi E, Tode T, Ishii K, Kudoh K and Nagata I (1993) Modulation of sensitivity of human ovarian cancer cells to cis-diamminedichloroplatinum (II) by 12-O-tetradecanoylphorbol-13-acetate and DL, buthionine-SR-sulphoximine. *Int J Cancer* **55**: 521–527

Hofmann J, Doppler W, Jakob A, Maly K, Posch L, Überall F and Grunicke HH (1988) Enhancement of the antiproliferative effect of cis-diamminedichoroplatinum (II) and nitrogen mustard by inhibitors of protein kinase C. *Int J Cancer* **42**: 382–388

Howell SB, Kirmani S, McClay EF, Kim S, Braly P and Plaxe S (1991) Intraperitoneal Cisplatin-based chemotherapy for ovarian carcinoma. *Semin Oncol* **18**: 5–11

Isonishi S, Andrews PA and Howell SB (1990) Increased sensitivity to cis-Diamminedichloroplatinum (II) in human ovarian carcinoma cells in response to treatment with 12-O-tetradecanoylphorbol 13-Acetate. *J Biol Chem* **265**: 3623–3627

Jozan S, Roche H, Cheutin F, Carton M and Salles B (1992) New human ovarian cancer cell line OVCCR1/Sf in serum free medium. *In Vitro Cell Dev Biol* **28A**: 687–689

Kurie JM, Younes A, Miller WH Jr, Burcher M, Chiu CF, Kolesnick R and Dmitrovsky E (1993*a*) Retinoic acid stimulates the protein kinase C pathway before activation of its beta nuclear receptor during human teratocarcinoma differentiation. *Biochim Biophys Acta* **1179**: 203–207

Kurie JM, Brown P, Salk E, Scheinberg D, Birrer M, Deutsch P and Dmitrowsky E (1993*b*) Cooperation between retinoic acid and phorbol esters enhances human teratocarcinoma differentiation. *Differentiation* **54**: 115–122

Le Ruppert KI, Masters JRW, Kneuchel R, Seegers S, Tainsky MA, Hofstaedter F and Buettner R (1992) The effect of retinoic acid on chemosensitivity of PA-1 human teratocarcinoma cells and its modulation by an activated N-*ras* oncogene. *Int J Cancer* **51**: 646–651

Mann SC, Andrews PA and Howell SB (1991) Modulation of cis-diamminedichloroplatinum (II) accumulation and sensitivity by forskolin and 3-isobutyl-1-methylxanthine in sensitive and resistant human ovarian carcinoma cells. *Int J Cancer* **48**: 866–872

Miller SA, Dykes DD and Polesky HF (1988) A simple salting out procedure for extracting DNA from human nucleated cells. *Nucleic Acids Res* **16**: 1215

Morikage T, Ohmori T, Nishio K, Fujiwara Y, Takeda Y and Saijo N (1993) Modulation of cisplatin sensitivity and accumulation by amphotericin B in cisplatin-resistant human lung cancer cell lines. *Cancer Res* **53**: 3302–3307

Nehme A, Albin N, Caliaro MJ, Guichard S, Jozan S, Julia AM, Bugat R and Canal P (1995) Mechanism of interaction between cisplatin and human recombinant interferon gamma in human ovarian-cancer cell lines. *Int J Cancer* **61**: 643–648

Nishikawa K, Rosenblum MG, Newman RA, Pandita TK, Hittelman WN and Donato NJ (1992) Resistance of human cervical carcinoma cells to tumor necrosis factor correlates with their increased sensitivity to cisplatin: evidence of a role for DNA repair and epidermal growth factor receptor. *Cancer Res* **52**: 4758–4765

Ozols RF and Young RC (1991) Chemotherapy of ovarian cancer. *Semin Oncol* **18**: 222–232

Reed E, Ozols RF, Tarone R, Yuspa SH, Poirier MC (1987) Platinum DNA-adducts in leucocytes DNA correlate with disease response in ovarian cancer patients receiving platinum-based chemotherapy. *Proc Natl Acad Sci USA* **84**: 5024–5028

Sacks PG, Harris D and Chou TC (1995) Modulation of growth and proliferation in squamous cell carcinoma by retinoic acid: a rationale for combination therapy with chemotherapeutic agents, *Int J Cancer* **61**: 409–415

Schiller U, Hofmann W, Mayer C, Ulrich W, Bamberg M and Rodemann HP (1994) All trans retinoic acid modulates the radiosensitivity and differentiation of normal and tumour cells, *in vitro*. *Ann Oncol* **5**: S3–S6

Sporn MB and Roberts AB (1983) Role of retinoids in differentiation and carcinogenesis. *Cancer Res* **43**: 3034–3040

Xia C, Taylor JB, Spencer SR and Kettener B (1993) The human gluthatione S transferase P1-1 gene: modulation of expression by retinoic acid and insulin. *Biochem J* **292**: 845–850

© Cancer Research Campaign 1997